# LINKS BETWEEN SOCIAL CONNECTEDNESS AND COGNITIVE HEALTH OPERATE THROUGH SOCIAL STIMULATION AND COGNITIVE RESERVE

**DOI:** 10.1093/geroni/igac059.952

**Published:** 2022-12-20

**Authors:** Siyun Peng, Anmoldeep Singh, Mohit Manchella, Brea Perry

**Affiliations:** Indiana University, Bloomington, Indiana, United States; Indiana University, Newburgh, Indiana, United States; University of Southern Indiana, Carmel, Indiana, United States; Indiana University, Bloomington, Indiana, United States

## Abstract

The link between social connectedness and dementia risk and resilience has been examined using a diverse set of measures. Though different measures of connectedness reflect distinct social processes and underlying mechanisms (e.g., stress buffering, cognitive stimulation), few studies have compared them. Using data from two social network studies of older adults (N=283), we compare associations between 29 measures of social connectedness and general cognitive function (MoCA), and non-verbal (Rey) and episodic memory (Craft). Measures of social participation (e.g., volunteering, working, attending church) and social support were unassociated with cognitive outcomes, net of controls. Quality of friendships (p<.05), family relationships (p<.01), and marriage (p<.05) were sporadically associated. Measures indicating large, diverse, and expansive networks were strongly and consistently related to all cognitive outcomes (e.g., number of phone contacts [p<.001], network size [p<.001], density [p<.001], racial homophily [p<.05], age heterogeneity [p<0.01], and diversity [p<.001]). We discuss implications for theories of cognitive reserve.

